# Genotypic variation in biomass allocation in response to field drought has a greater affect on yield than gas exchange or phenology

**DOI:** 10.1186/s12870-016-0876-3

**Published:** 2016-08-24

**Authors:** Christine E. Edwards, Brent E. Ewers, Cynthia Weinig

**Affiliations:** 1 Department of Botany, University of Wyoming, Laramie, WY 82071 USA; 2 Current Address: Center for Conservation and Sustainable Development, Missouri Botanical Garden, PO Box 299, St. Louis, MO 63166 USA; 3 Program in Ecology, University of Wyoming, Laramie, WY 82071 USA; 4 Department of Molecular Biology, University of Wyoming, Laramie, WY 82071 USA

**Keywords:** *Brassica rapa*, Genotype by environment interactions, Drought escape, Dehydration avoidance, QTL

## Abstract

**Background:**

Plant performance in agricultural and natural settings varies with moisture availability, and understanding the range of potential drought responses and the underlying genetic architecture is important for understanding how plants will respond to both natural and artificial selection in various water regimes. Here, we raised genotypes of *Brassica rapa* under well-watered and drought treatments in the field. Our primary goal was to understand the genetic architecture and yield effects of different drought-escape and dehydration-avoidance strategies.

**Results:**

Drought treatments reduced soil moisture by 62 % of field capacity. Drought decreased biomass accumulation and fruit production by as much as 48 %, whereas instantaneous water-use efficiency and root:shoot ratio increased. Genotypes differed in the mean value of all traits and in the sensitivity of biomass accumulation, root:shoot ratio, and fruit production to drought. Bivariate correlations involving gas-exchange and phenology were largely constant across environments, whereas those involving root:shoot varied across treatments. Although root:shoot was typically unrelated to gas-exchange or yield under well-watered conditions, genotypes with low to moderate increases in root:shoot allocation in response to drought survived the growing season, maintained maximum photosynthesis levels, and produced more fruit than genotypes with the greatest root allocation under drought. QTL for gas-exchange and yield components (total biomass or fruit production) had common effects across environments while those for root:shoot were often environment-specific.

**Conclusions:**

Increases in root allocation beyond those needed to survive and maintain favorable water relations came at the cost of fruit production. The environment-specific effects of root:shoot ratio on yield and the differential expression of QTL for this trait across water regimes have important implications for efforts to improve crops for drought resistance.

**Electronic supplementary material:**

The online version of this article (doi:10.1186/s12870-016-0876-3) contains supplementary material, which is available to authorized users.

## Background

Drought stress leads to significant reductions in both yield in crops and fitness in wild plants species. Water availability is unpredictable in many regions of the world and is expected to become increasingly unpredictable under ongoing climate change [1]. As a consequence, characterizing the genetic range of potential drought responses, identifying genotypes with adaptive drought responses, and predicting how crops will perform under global climate change are among the primary aims of current crop research [2–8].

Plants acclimate to environmental stress through a combination of physiological adjustments during the course of a single day and longer-term plasticity over days to months. Some plastic responses may allow plants to avoid dehydration when faced with water deficits [9–11]. For example, in response to drought, plants close their stomates; this response minimizes water loss from transpiration, but also decreases rates of stomatal conductance, photosynthesis, and growth [11]. Plasticity in other traits such as relative biomass allocation to roots versus above-ground organs frequently enables greater water uptake in mild drought and survival in severe drought conditions [12, 13], but may likewise reduce the harvestable component in crops. Other responses allow for drought escape, such as shifts in phenology that enable plants to complete their lifecycle rapidly and elude drought stress altogether [9, 10, 14]; phenological acceleration, however, limits the time available to grow prior to reproduction and may thereby reduce yield. Within a species, genotypes may harbor different alleles or show allelic sensitivity at causal loci, leading to differential responses to environmental stress (i.e., genotype × environment interactions). Those genotypes with greater average performance across soil moisture levels or with adaptive phenotypic responses that minimize tradeoffs with yield can provide a foundation for crop improvement to increase yield in drought conditions.

Genetic correlations, arising from either pleiotropy or close physical linkage of genes encoding different traits, may limit adaptation and crop improvement if the major axis of trait covariation is counter to the joint vector of selection on agronomically desirable traits [15]. For instance, selection by breeders may favor increased root:shoot ratios in combination with somewhat reduced stomatal conductance under low water availability (i.e., selection favors a negative correlation), but the response to selection will be weak if the correlation between these two traits is positive (i.e., selection to increase the value of the first trait would lead to a correlated and undesired increase in the value of the second trait) [16–18]. Because different genes may affect phenotypic traits in different environments or functional differences between alleles may vary across environments, the expression of genetic variation and the patterns of covariation among traits may change across settings [19–24], such that environmental heterogeneity also influences the response to selection [25]. Correlations between the expression of a single trait across two environments (e.g., root:shoot under well-watered *vs*. drought conditions) may likewise affect the opportunity for adaptive evolution or crop improvement. Thus, to understand how specific crops will respond to improvement efforts, it is important to quantify the relative magnitude of genotype and genotype × environment interaction variances as well as genetic correlations among traits and the environmental dependency of these correlations [18, 25–27].

Responses to drought are complex, involving diverse gas-exchange, allocation and phenological traits. As alluded to above, the agronomic value of selective breeding for either a drought-escape or dehydration-avoidance strategy likely depends on the magnitude and duration of the drought stress and on possible yield tradeoffs associated with drought responses, yet a comprehensive examination of the genetic architecture associated with these diverse drought-response strategies and their yield effects in the field is largely lacking. In this study, we investigated the genetic architecture of diverse drought responses in *Brassica rapa* L., a plant species cultivated worldwide as a vegetable and oilseed crop. The genetic architecture of drought-response traits in *B. rapa* was investigated previously in a greenhouse experiment that revealed significant changes in the correlations between water-use efficiency and plant performance traits across treatments as well as a negative across-environment correlation for water-use efficiency [24]. The study further revealed a subset of genotypes that optimally matched their water-use efficiency to the environment, resulting in greater biomass and gas exchange across both drought and well-watered conditions [24]. However, the results of greenhouse studies may not always translate to field conditions [28–30], due to the complexity of field settings, simultaneous variation in many environmental factors, and divergent yield responses. Here, our goals were to understand: 1) which specific traits, such as stomatal conductance, water-use efficiency, allocation, phenology, etc., are responsive to and maximize yield under season-long field drought in *B. rapa*, 2) if different or similar genotypes perform best in drought and well-watered conditions, 3) whether moisture status in the field affects the magnitude and direction of genetic correlations between mechanistically-related (e.g., gas-exchange traits) or -unrelated (e.g., gas-exchange traits and phenology) drought-response traits, and 4) patterns of QTL effects across water regimes, including allelic contributions from parental genotypes with divergent selection histories.

## Methods

### Study species and plant material

*Brassica rapa* is an oilseed and vegetable crop species whose original range of cultivation extends from the western Mediterranean to Central Asia [31]. Crops of *B. rapa* include varieties cultivated as oilseeds (*B. rapa* subsp. *oleifera*, or rapeseed oil), root vegetables (*B. rapa* subsp. *rapa*, or turnip), and leafy vegetables (*B. rapa* subsp. *chinensis*, or pak choi, and *B. rapa* subsp. *pekinensis*, or Chinese cabbage). The species also occurs commonly in naturalized populations in proximity to crop fields [32].

In the present study, we used 121 recombinant inbred lines that resulted from a cross between two inbred genotypes of *B. rapa*, R500 and IMB211 [33]. The IMB211 genotype was derived from the Wisconsin Fast Plant™ population; artificial selection for rapid generation time in IMB211 resembles that experienced by naturalized populations and agricultural weeds of this species [32, 34]. The R500 genotype is a seed-oil cultivar planted in India for at least 3,000 years [35]. Given their divergent selection histories, genetic variation segregating in the RILs may resemble that segregating in crop × wild hybrids found commonly in nature [36], and the RILs are expected to harbor increased diversity beyond many cultivated lines. Furthermore, the parents of the RILs differ in life history, vegetative, reproductive and leaf gas-exchange traits [37–41], suggesting that this is a relevant population in which to investigate the genetic architecture of drought responses.

### Experimental design

The experiment was carried out at the University of Wyoming Agricultural Experiment Station in Laramie, WY from June through September, 2010. For each treatment (drought and well-watered), we planted ten replicates of each of the 121 RILs and the two parents (*n* = 123 genotypes × 10 replicates × 2 treatments = 2460 individuals total). Plants in each treatment were arranged into ten blocks, each containing one individual of each of the 123 genotypes in a completely randomized design. Seeds were planted on June 8-9, 2010 in two greenhouse bays, with the number of blocks of each treatment equally represented in each greenhouse bay. For each replicate plant, three seeds were planted in ~680 ml peat pots (Jiffy products of America, Lorain, OH, USA) containing 2 ml of Osmocote 18-6-12 fertilizer (Scotts Miracle Grow, Marysville, OH, USA) and field soil (autoclaved to prevent germination of non-target plant species). Field soil at the Wyoming Agricultural Experiment Station is characterized as Wycolo-Alcova complex (3-10 % slopes), a stratified mixture of reddish brown fine loam, brown sandy loam, and reddish brown clay [42]. Seeds were allowed to germinate in the greenhouse under moist soil conditions, during which time germinants were thinned to one seedling closest to the center of the pot. Plants received 16 h/8 h light/dark natural light cycles in the greenhouse, with temperatures fluctuating diurnally from 18 – 30 °C to match ambient conditions outdoors. After germinating for 15 days, plants were developing their first true leaf and were transplanted in the field on June 23–24, 2010. Plants were arranged into prepared blocks with 25 cm between replicates, a distance great enough to forestall potential shade-avoidance responses in this species [43].

Treatments were imposed two days after transplanting. For all plants, the volumetric water content (VWC) of the soil was monitored throughout the experiment using a 10-HS soil moisture meter with an ECH_2_O Check analog read-out system (Decagon Devices, Pullman, WA, USA), which measures soil moisture in a ~1.3 L volume surrounding the sensor. Measurements were taken in the uppermost 10 cm of soil. Plants in the well-watered treatment were irrigated twice daily for 30 min, which maintained moist soil conditions. For the drought treatment, our goal was to impose drought conditions similar to those experienced in agricultural settings that cause losses in yield without leading to mortality; almost no experimental plants (<10) died following field transplanting, but fruit production was significantly reduced (see Results). Plants in the drought treatment were watered briefly by hand when VWC measurements taken throughout a block were ≤10 %. The average VWC at flowering was 6.5 % in the drought treatment and 17.1 % in the well-watered treatment.

### Trait measurements

Plants were checked daily for flowering (i.e., when the sepals opened and petals became visible), at which time the number of days from planting to flowering was scored. Flowering began July 2, 2010. Leaf gas exchange was measured at flowering using a steady-state gas-exchange system equipped with a leaf chamber fluorometer (LICOR-6400XT; LI-COR Biosciences Inc., Lincoln, NE, USA). Because of greater within-genotype variation in gas-exchange traits, measurement of gas-exchange traits was carried out on all 10 replicates per genotype, whereas all other traits were measured on eight out of the 10 replicates per genotype due to time constraints. Measurements were taken on a young, fully expanded leaf, but avoiding the first true leaf to ensure that the leaf developed entirely in the field. We measured photosynthesis (*A*), chlorophyll fluorescence in light (*F*_*v*_*′/F*_*m*_*',* or maximum photosystem II efficiency in light, a key measurement of the light-dependent reactions of photosynthesis), and stomatal conductance (*g*_*s*_), as described previously [24, 41]. Leaf cuvette conditions were set to a photosynthetic irradiance of 2000 μmol m^-2^ s^-1^ to measure the maximum photosynthetic rate of the plants, with incoming air CO_2_ concentration set to 400 μmol mol^-1^, and leaf temperature maintained at 26 °C to match daytime temperature conditions in the field. These measurements were used to calculate intrinsic water-use efficiency (*W*_*g*_) for each individual by dividing *A* by *g*_*s*_ [44]. The leaf was then removed, scanned, oven dried, and weighed. Leaf area was measured from the scanned leaf images using ImageJ [45] and used to calculate leaf mass per area (LMA; g m^-2^), which is mechanistically related to both photosynthetic gas supply and biochemical demand [46, 47].

Carbon isotope (δ^13^C) composition, a time-integrated estimate of *W*_*g*_, was analyzed on a composite sample of the eight individuals of each genotype in each treatment. The oven-dried leaves collected at bolting were ground and pooled in equal weights for isotope analysis. Carbon isotope composition was analyzed using an elemental analyzer (ECS 4010, Costech Analytical Technologies, Inc., Valencia, CA, USA) coupled to a continuous-flow inlet isotope ratio mass spectrometer (CF-IRMS; Delta-plus XP, Thermo Scientific, Waltham, MA, USA). δ^13^C values were reported in parts per thousand relative to Vienna Peedee Belemnite (VPDB). The precision of repeated measurements of laboratory standards was <0.1‰. All stable isotope analyses were performed at the University of Wyoming Stable Isotope Facility. We assume that δ^13^C of the air was constant in these experiments because they were conducted in the field; thus, because measurements of δ^13^C of a leaf are directly proportional to carbon isotope discrimination (∆^13^C) under uniform δ^13^C_air,_ we only present isotope results as δ^13^C.

Plants were harvested when flowering finished and plants ceased to develop new fruits. At harvest, which occurred on August 31-September 1, 2010, the above-ground height of the plants and number of fruits was recorded, and the above-ground biomass and taproot [a proxy for total belowground biomass, 39] were removed, oven dried, and weighed. We estimated the root:shoot ratio to provide an assessment of relative biomass allocation.

### Quantitative genetic analyses

SAS ver. 9.2 was used for all ANOVA and correlation analyses. In each treatment, we used restricted maximum likelihood (REML in PROC MIXED) to estimate the random effects of genotype and block on each phenotypic trait. Because we used four LI-COR 6400XT machines to measure *A*, *g*_*s*_, *F*_*v*_*′/F*_*m*_*'*, and *W*_*g*_, we included an identifier for the machine (IRGA ID) as an additional random factor in the analyses of these traits. The table-wise significance values for these ANOVAs were corrected for multiple comparisons by controlling the false discovery rate at α = 0.05 (FDR, [48]). The variance components estimated from this analysis were used to estimate the ratio *V*_*G*_*/V*_*P*_, or broad-sense heritability, where *V*_*G*_ is the among-genotype variance component in each treatment and *V*_*P*_ is the sum of all variance components for a trait in each treatment. For all traits, this analysis was used to estimate the genotypic values of each trait as best linear unbiased predictors (BLUPs, [49]). The treatment mean was added to the BLUPs for each trait, such that the genotypic means reflect the actual scale of each trait. These means were used for subsequent Pearson correlations and QTL analysis.

We tested for genotypic differences in the response to treatment using a mixed-model nested ANOVA across the two treatments (PROC MIXED, SAS ver. 9.2). We evaluated the fixed effect of treatment and the random effects of genotype, block nested within treatment, and the genotype × treatment interaction on each trait. Table-wise FDR correction was again used to control for multiple tests.

To estimate genetic correlations among traits within each treatment, we performed a multivariate ANOVA using restricted maximum likelihood, which takes measurement error into account [50, 51] (SAS PROC mixed). To assess whether these correlations were significantly different than 0, we used a likelihood ratio test to compare a model in which correlations were unconstrained against one in which correlations were constrained to 0 [50, 52]. We also used point estimates of the genotypic values (as BLUPs) of each trait in each treatment to estimate bivariate Pearson product-moment correlation coefficients among traits (SAS PROC CORR). Correlation coefficients using the ANOVA approach were uniformly larger but proportional to results using the bivariate approach; we thus show only the results of the bivariate correlation analyses (Table 4). The significance values of all bivariate correlations were corrected for multiple comparisons by controlling the FDR. We used Fisher’s Z-tests [53] to identify bivariate correlations that were significantly different across treatments for all pairs of traits.

To further assess how the genetic architecture of each trait differed across treatments, we estimated *r*_*GE*_, the genotypic correlation of each trait across the two treatments [54–56]. First, we used the multivariate ANOVA approach described above to estimate correlations for each trait across the two treatments [50, 51] (SAS PROC mixed). We also used point estimates of the genotypic values of each trait in each treatment (as BLUPs) to estimate Pearson product-moment correlation coefficients for each trait across treatments (SAS PROC CORR). For the reasons given above, we present only the results of the bivariate Pearson correlations. Estimates of *r*_*GE*_ indicate the extent to which the same genetic loci are expressed and allele pairs have the same functional effects across treatments; estimates of *r*_*GE*_ approaching 1 suggest that the genetic basis of the trait is similar across environments, whereas estimates approaching 0 suggest that different genetic loci affect the trait or alleles at a locus differ in their functional relationship across treatments [54–56]. ANOVA for each trait across the two treatments were used to determine the significance of *r*_*GE*_; *r*_*GE*_ is significantly less than 1 when the genotype × treatment interaction is significant, and significantly different from 0 when the among-genotype variance is significant [54, 55].

### QTL mapping

The linkage map used in this study was a highly resolved RNA-seq based SNP map with 1273 informative genomic bins (“markers”) distributed across the 10 *B. rapa* chromosomes with an average distance of 0.79 cM [57, 58]. Genotypic bins were delineated by genotyping 124 RILs at >65 K SNP positions. SNPs were identified by a samtools/bcftools-based analysis using >355 million mapped 44 bp RNA-seq reads with an average depth across the transcriptome of 2.6 reads per RIL. Since only a fraction of genes are expressed, the actual coverage for expressed genes is significantly greater. QTL mapping of each trait in each treatment was carried out using composite interval mapping as implemented in Windows QTL Cartographer ver. 2.5 [59] following the methodology described in Edwards and Weinig [37]. The genome-wide significance threshold was determined for each trait using 1000 permutations [60] with a type-I error rate of 0.05.

We tested for significant differences in QTL effects for each trait across environments using single-marker analysis of variance [61] (PROC GLM, SAS ver. 9.2). In the single-marker analysis, the model tested the fixed effects of treatment, the genotype at the marker nearest to each detected QTL, and the marker × treatment interaction on the genotypic values of each trait.

## Results

### Results of ANOVA

Within both the well-watered (WW) and drought (DR) treatments, we carried out analysis of variance for each trait to test the random effects of genotype and block (Table 1). Because we used four different infrared gas analyzer instruments, we also included this as a random effect in the analyses of gas-exchange traits; however, we did not find a significant effect of instrument for any of the four gas-exchange traits, and we do not report these effects further. All traits demonstrated significant among-genotype variance in both treatments (*P* < 0.001; Table 1). In both treatments, estimates of broad sense heritability (*V*_*G*_*/V*_*P*_) varied across traits. *V*_*G*_*/V*_*P*_ was low (≤0.25) in both treatments for photosynthesis (*A*), stomatal conductance (*g*_*s*_), leaf mass per area (LMA) and root:shoot ratio. *V*_*G*_*/V*_*P*_ was moderate to high for the remaining traits, with the greatest values found for δ^13^C (0.64 and 0.75 in WW and DR, respectively), fruit production (0.57 in both treatments), and chlorophyll fluorescence in light (*Fv'/Fm'*) (0.54 and 0.67 in WW and DR, respectively).

**Table 1:** Quantitative genetic partitioning and significance of effects for leaf gas-exchange, vegetative, and reproductive traits for genotypes of *B. rapa* within the well-watered (WW) and drought (DR) treatments. Standard errors are indicated in parentheses

Trait	WW V_G_ (SE)	WW block effect (SE)	WW V_G_/V_P_	DR treatment V_G_ (SE)	DR block effect (SE)	DR V_G_/V_P_
*A* (μmol m^-2^ s^-1^)	21.10 (3.71)**	1.16 (0.88) ^NS^	0.21	25.24 (4.66)**	1.09 (0.96) ^NS^	0.24
*Fv'/Fm'*	0.004 (0.00)**	0.000 (0.000) ^NS^	0.54	0.005 (0.0007)**	0.00003 (0.00003) ^NS^	0.67
g_s_ (mol m^-2^ s^-1^)	0.003 (0.0008)**	0.001 (0.001) ^NS^	0.09	0.004 (0.0009)**	0.0009 (0.0006) ^NS^	0.14
*W*_*g*_	34.49 (16.63)**	6.79 (6.96) ^NS^	0.05	60.72 (17.20)**	10.29 (7.59) ^NS^	0.14
δ^13^C	1.29 (0.49)**	0.004 (0.04) ^NS^	0.64	1.31 (0.4835)**	0.05 (0.05) ^NS^	0.75
LMA (g m^-2^)	29.57 (8.52)**	1.77 (2.23) ^NS^	0.13	17.641 (7.08)**	3.02 (3.03) ^NS^	0.09
Above-ground biomass (g)	118.53 (17.46)**	30.05 (16.6)^‡^	0.46	42.52 (6.12)**	4.84 (2.74)^‡^	0.57
Below-ground biomass (g)	0.16 (0.02)**	0.02 (0.008)^‡^	0.48	0.06 (0.008)**	0.005 (0.003)^‡^	0.61
Root:Shoot ratio	0.00003 (0.000008)**	0.00002 (0.00001)^‡^	0.13	0.0001 (0.00002)**	0.00002 (0.00001) ^NS^	0.20
Leaf area (cm^2^)	39.14 (6.66)**	4.38 (3.13) ^NS^	0.36	10.96 (2.39)**	0.47 (0.49) ^NS^	0.25
Plant height (cm)	103.84 (15.65)**	4.30 (2.87) ^NS^	0.46	84.3845 (15.0446)**	6.21 (4.31) ^NS^	0.31
Fruit production (number of fruits)	10672 (1502.06)**	526.17 (317.08)^‡^	0.57	3095.72 (441.96)**	206.77 (121.81)^‡^	0.57
Days to flowering	3.91 (0.66)**	0.57 (0.34)^‡^	0.30	4.76 (0.77)**	0.23 (0.16) ^NS^	0.39

*V*_*G*_ among-genotypic variance, *V*_*G*_ */V*_*P*_ among-genotypic variance divided by total phenotypic variance, *A,* photosynthetic rate, *Fv'/Fm'* chlorophyll fluorescence in light, *g*_*s*_ stomatal conductance, *W*_*g*_ intrinsic water use efficiency (*A*/*g*_*s*_), *δ* ^*13*^ *C* carbon isotope composition, *LMA* leaf mass per area, ^‡^ *P* < 0.05, **P < 0.0001, ^NS^ not significant

Across treatments, we partitioned variance attributable to block(treatment), genotype, treatment, and the genotype × treatment interaction using a mixed-model nested ANOVA. Water regime had a significant effect on the expression of 9 of the 13 traits investigated in this study. Plants in the DR treatment had greater intrinsic water use efficiency (*W*_*g*_) than in the WW treatment (Tables 2 and 3), which resulted from plants decreasing *g*_*s*_ in the DR treatment while maintaining similar average photosynthetic rates in the two treatments. Relative to plants in the WW treatment, plants in the DR treatment had significantly lower above-ground and below-ground biomass and a 24 % greater root:shoot ratio (Tables 2 and 3), indicating that plants experiencing drought stress were smaller but allocated proportionally more biomass to roots than to shoots. Plants in the DR treatment also had significantly larger LMA, smaller leaf area, were significantly shorter, and produced an average of 48 % fewer total fruits (Tables 2 and 3).

**Table 2:** Treatment means across RILs and for each parent for leaf gas-exchange, vegetative, and reproductive traits for genotypes of *B. rapa* within the well-watered (WW) and drought (DR) treatments. Standard errors are indicated in parentheses

Trait	WW RIL treatment mean (SE)	WW IMB211 parent mean	WW r500 parent mean	DR RIL treatment mean (SE)	DR IMB211 parent mean	DR r500 parent mean
*A* (μmol m^-2^ s^-1^)	27.50 (2.85)	25.09	28.40	26.52 (2.33)	25.09	27.72
*Fv'/Fm'*	0.45 (0.02)	0.47	0.48	0.44 (0.02)	0.46	0.45
g_s_ (mol m^-2^ s^-1^)	0.43 (0.04)	0.36	0.42	0.37 (0.03)	0.32	0.39
*W*_*g*_	69.33 (1.38)	74.08	70.12	76.86 (1.52)	77.0	75.09
δ^13^C	-28.03 (0.28)	NA	NA	-27.87 (0.29)	NA	NA
LMA (g m^-2^)	58.97 (0.91)	57.55	55.71	62.04 (0.97)	59.0	61.11
Above-ground biomass (g)	19.32 (2.21)	9.77	67.48	10.09 (1.00)	5.66	40.61
Below-ground biomass (g)	0.54 (0.06)	0.30	2.78	0.37 (0.03)	0.35	1.60
Root:Shoot ratio	0.029 (0.00)	0.032	0.036	0.04 (0.002)	0.063	0.041
Leaf area (cm^2^)	14.99 (1.07)	13.11	48.48	11.89 (0.47)	11.55	20.89
Plant height (cm)	39.46 (1.23)	26.87	72.85	33.16 (1.31)	24.76	60.95
Fruit production (number of fruits)	179.83 (12.72)	143.03	306.78	93.06 (7.35)	75.60	198.67
Days to flowering	34.91 (0.34)	31.63	38.91	34.51 (0.28)	31.38	39.48

*A* photosynthetic rate, *Fv'/Fm'* chlorophyll fluorescence in light, *g*_*s*_ stomatal conductance, *W*_*g*_ intrinsic water use efficiency (*A*/*g*_*s*_), *δ* ^*13*^ *C* carbon isotope composition, *LMA* leaf mass per area

**Table 3:** Quantitative genetic partitioning of variation and significance of effects across drought and control treatments, and Pearson correlation coefficients for across-treatment genotypic correlations (*r*_*GE*_) for each trait. Standard error is indicated in parenthesis. Estimates of *r*_*GE*_ for all traits are significantly different than 0, as indicated by a significant effect of Genotype; *r*_*GE*_ for above- and below-ground biomass, root:shoot ratio, and fruit production are significantly less than 1, as indicated by significant effects of genotype × treatment interactions

Random effects						Fixed effect		Cross env corr.
Trait	Block(treatment)	Genotype	Genotype × treatment	IRGA ID	Residual	Treatment		*r*_*GE*_
						df	F	
*A*	1.0489 (0.6012)^‡^	24.6608 (3.7907)**	0 (0)^NS^	25.2078 (20.6757) ^NS^	52.363 (1.9286)**	F_1, 13.9_	1.83^NS^	0.79
*Fv'/Fm'*	0.00004 (0.00002)^‡^	0.004 (0.0006)**	0.000004 (0.00004) ^NS^	0.001 (0.0008) ^NS^	0.002 (0.00007)**	F_1, 13.9_	0.10^NS^	0.94
g_s_ (mol m^-2^ s^-1^)	0.0008 (0.0004)^‡^	0.004 (0.0007)**	0 (0)^NS^	0.005 (0.004) ^NS^	0.02 (0.0007)**	F_1, 13.9_	10.52^§^	0.59
*W*_*g*_	8.147 (5.031)^†^	48.858 (11.887)**	0 (0)^NS^	0 (.)^NS^	516.8 (19.030)**	F_1, 14_	16.4^§^	0.37
δ^13^C	0.032 (0.032) ^NS^	1.361 (0.4875)^§^	0 (0)^NS^	--	0.532 (0.063)**	F_1, 10.3_	0.84^NS^	0.83
LMA (g m^-2^)	2.270 (1.813)^NS^	27.552 (6.051)**	0 (0)^NS^	--	182.32 (7.717)**	F_1, 9.44_	6.88^‡^	0.49
Above-ground biomass (g)	16.732 (6.578)^§^	76.132 (11.384)**	10.403 (2.784)*	--	69.470 (2.678)	F_1, 15.2_	18.73*	0.89
Below-ground biomass (g)	0.009 (0.004)	0.099 (0.015)**	0.012 (0.004)*	--	0.098 (0.004)	F_1, 16.5_	12.09^§^	0.90
Root:Shoot ratio	0.00002 (0.000009)^‡^	0.00004 (0.00001)**	0.00003 (0.00001)^§^	--	0.0003 (0.00001)**	F_1, 16.2_	21.57*	0.42
Leaf area (cm^2^)	2.393 (1.271)^‡^	25.084 (4.440)**	2.280 (1.798) ^NS^	--	48.864 (2.182)**	F_1, 11_	9.76^§^	0.70
Plant height (cm)	5.308 (2.570)^‡^	94.227 (13.967)**	2.292 (3.182) ^NS^	--	148.64 (5.635)**	F_1, 14.4_	22.41*	0.80
Fruit production (number of fruits)	384.17 (162.83)^§^	5832.09 (887.96)**	1227.68 (251.17)**	--	4857.45 (183.6)**	F_1, 19.7_	59.06**	0.90
Days to flowering	0.3736 (0.166)^‡^	4.074 (0.596)**	0 (.)^NS^	--	6.8388 (0.243)**	F_1, 13.9_	1.88^NS^	0.74

*A* photosynthetic rate, *Fv'/Fm'* chlorophyll fluorescence in light, *g*_*s*_ stomatal conductance, *E* transpiration rate, *W*_*g*_ intrinsic water use efficiency (*A*/*g*_*s*_), *δ* ^*13*^ *C* carbon isotope composition, *N*_*area*_ nitrogen concentration on a leaf area basis, *LMA* leaf mass per area, ^NS^not significant, ^†^ *P* < 0.1,^‡^ *P* < 0.05, ^§^ *P* < 0.01, ^*^ *P* < 0.001,^**^ *P* < 0.0001

Only 4 of the 13 traits investigated in this study demonstrated significant genotype × treatment interactions (i.e., genotype × environment interactions; G × E; Table 3), including above-ground biomass, below-ground biomass, root:shoot ratio, and fruit production.

### Results of genetic correlations among traits

To assess the relationship among traits, we estimated genotypic correlations between trait pairs. With several notable exceptions that are discussed below, the magnitude and direction of most bivariate correlations between trait pairs were not significantly different across treatments. Photosynthesis (*A*) was significantly correlated with many other traits. *A* was positively correlated with *Fv'/Fm'* (Table 4), a measure of the efficiency of the light-harvesting reactions. Because of the strong positive association of *A* with *Fv'/Fm'*, patterns of correlations were similar for these two traits. *A* and *Fv'/Fm'* were positively correlated with δ^13^C in both treatments and with other traits involved in water use, such as *g*_*s*_ and *W*_*g*_ (Table 4), indicating that a higher photosynthetic rate and higher efficiency of light-harvesting reactions were associated with greater rates of water use and water-use efficiency. *A* and *Fv'/Fm'* were also both positively correlated with LMA and other vegetative traits, such as above-ground biomass, below-ground biomass, leaf area, plant height and total fruit production (Table 4), indicating that genotypes with a greater photosynthetic rate and greater efficiency of light .09pt?>harvesting reactions were larger and had greater fruit production under both drought and well-watered conditions.

**Table 4:**
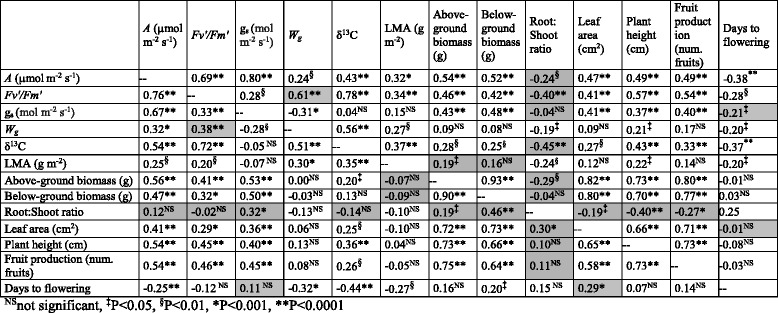
Pearson correlation coefficients and significance of bivariate genetic correlations among traits. Values above the diagonal indicate genetic correlations among traits in the drought treatment and values below the diagonal indicate genetic correlations among traits in the well-watered treatment. Symbols denote the significance of correlations after false discovery rate correction (*P* < 0.05) and correlations shaded in gray indicate those for which Z-tests found significant differences in correlation coefficients across treatments

Stomatal conductance (*g*_*s*_) was also significantly correlated with additional traits beyond photosynthesis. *g*_*s*_ was negatively correlated with *W*_*g*_ (Table 4), indicating that genotypes with greater rates of water loss were less water-use efficient. However, *g*_*s*_ was uncorrelated with δ^13^C (Table 4). With the exception of LMA (which was uncorrelated with *g*_*s*_), *g*_*s*_ was positively correlated with most plant performance and fitness traits in both treatments, including above-ground biomass, below-ground biomass, leaf area, plant height, and fruit production (Table 4), indicating that plants with greater rates of water use (and consequently greater photosynthesis) accumulate more biomass and have greater fruit production.

The phenological trait, days to flowering, was significantly negatively correlated with *A*, *W*_*g*_, δ^13^C, and LMA (Table 4), indicating that early-flowering genotypes have a greater photosynthetic rate and water-use efficiency. Days to flowering was uncorrelated or weakly correlated with above- and below-ground biomass, root:shoot ratio, plant height, and fruit production (Table 4). Vegetative and plant performance traits, such as above-ground biomass, below-ground biomass, leaf area, plant height, and fruit production, were all strongly positively correlated with each other in both treatments (Table 4).

**Fig. 1:**
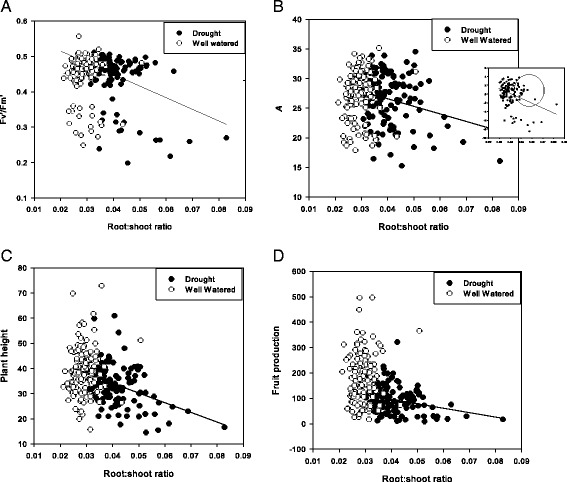
Comparisons between genotypic correlations in drought (solid black circles) and well - watered conditions (open white circles) between root:shoot ratio and **a** chlorophyll fluorescence in light (*Fv'/Fm'*), **b** photosynthetic rate, *A*, **c** plant height, and **d** fruit production. Regression lines are shown for significant correlations, which occurred only in the drought treatment. The inset in **b** shows the residuals of *A* in drought after accounting for g_s_ versus root:shoot in drought, with the circle indicating genotypes that maintain a high level of photosynthesis together with a moderate value of root:shoot

In contrast to the trait associations listed above that involve gas-exchange traits, several bivariate correlations demonstrated significant differences across treatments, primarily involving root:shoot ratio but also days to flowering (see shaded cells, Table 4). Root:shoot ratio was uncorrelated with *A, Fv'/Fm'*, δ^13^C, *W*_*g*_, LMA, plant height, and fruit production in the WW treatment and significantly negatively correlated with these same traits in DR treatment (Table 4, Fig. 1a-c). Root:shoot ratio was also positively correlated with *g*_*s*_, leaf area, and above- and below-ground biomass in the WW treatment, and either uncorrelated (in the case of *g*_*s*_) or negatively correlated (in the case of leaf area and biomass) with these traits in DR (Table 4). These results indicate that greater allocation to roots *vs*. shoots among genotypes is unrelated to photosynthesis or yield in well-watered conditions. Under drought conditions, genotypes with intermediate values of root:shoot had *Fv'/Fm'* and *A* that were similar to those observed in well-watered conditions (Fig. 1a and b), but genotypes that had the greatest allocation to roots relative to shoots had lower photosynthesis, reduced vegetative size, and lower fruit production. Further, genotypes in the WW treatment with greater proportional allocation of biomass to roots had larger roots and greater water use, whereas proportional allocation of biomass was unrelated to root biomass and overall water loss in the DR treatment.

Days to flowering showed weak evidence of shifts in correlation with two traits across treatments. Days to flowering was uncorrelated with *g*_*s*_ in the WW treatment, but these traits were significantly negatively correlated in the DR treatment, indicating that genotypes with greater stomatal conductance flowered earlier under drought but not well-watered conditions. Days to flowering was significantly positively correlated with leaf area in the WW treatment whereas these traits were uncorrelated in the DR treatment, indicating that vegetatively large genotypes delayed flowering in the WW but not DR treatment.

### Results of across-environment genetic correlations

To further investigate whether the genetic architecture of traits varied with moisture status, we estimated genotypic correlations across treatments. Overall, values of *r*_*GE*_ ranged from 0.37 to 0.94. The lowest values were found for *W*_*g*_ (0.37), root:shoot ratio (0.42), and LMA (0.49). For most traits (*A*, *Fv'/Fm'*, *g*_*s*_, *W*_*g*_, δ^13^C, LMA, leaf area, plant height, and days to flowering), *r*_*GE*_ estimates were significantly different than 0 (i.e., significant effect of genotype) and not or only moderately (*P* < 0.01) significantly different from 1 (i.e., non-significant G × E; Table 3), indicating that common loci affected the trait across treatments and that alleles at causal loci had similar functional relationships across the treatments. For most allocation traits (above-ground biomass, below-ground biomass, root:shoot ratio, and fruit production), estimates of *r*_*GE*_ were significantly different than both 0 and 1 (that is, both genotype and G × E effects were highly significant; Table 3), indicating that different loci affected the trait across treatment pairs and/or that some alleles had different functional effects across treatments.

### QTL analysis

**Fig. 2:**
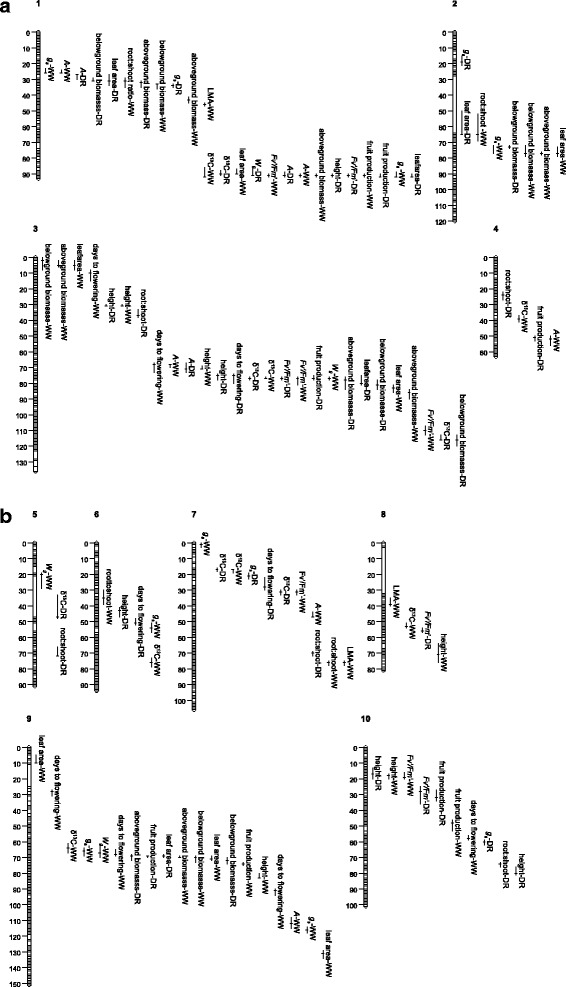
QTL detected for traits measured in drought (DR) and well-watered (WW) treatments. The length of bars designate the range of 2-LOD support limits for each QTL, with the peak of the QTL indicated. **a** QTL on chromosomes 1-5, and **b** QTL on chromosomes 6-10

In the genome-wide scans for main-effect QTL we found a total of 116 significant QTL that were detected on all 10 chromosomes (Table 5; Fig. 2). 66 of the QTL were detected in the WW treatment, and 50 were detected in the DR treatment. Below, we highlight QTL results of relevance to drought responses and the potential for either correlated or independent responses to selection.

**Table 5:** Results of composite interval QTL mapping and QTL × environment interactions of traits in *Brassica rapa* RILS. The trait and treatment for which the QTL was detected, chromosomal position (in cM), 2-LOD support intervals (in cM), additive effect with respect to the IMB211 allele, and percent variance explained are listed. The closest marker for each QTL is listed, with markers named with physical positions of SNPs relative to the *B. rapa* genome version 1.5 (available at BRAD). QTL are organized by cM position of the QTL peak for each chromosome in accordance with their position in Fig. 2. The *P*-value of QTL that demonstrate significant QTL × E interactions (*P* < 0.05) across treatments are indicated

Trait/treatment	Position in cM (2-LOD intervals)	Additive effect	% variance explained	Closest marker	*P*-value of QTL × E (*P* < 0.05)
Chromosome 1					
*g*_*s*_-WW	25.6 (22.5-26.4)	0.010	7.7	A01x7382304	
*A*-WW	25.6 (24.2-26.4)	0.91	4.6	A01x7382304	
*A*-DR	27.3 (26.4-29.6)	1.58	11.8	A01x8029418	
belowground biomass-DR	30.5 (29.3-31.3)	0.082	15.8	A01x8409488	
leaf area-DR	30.5 (26.9-34.2)	0.67	6.8	A01x8409488	
root:shoot ratio-WW	30.5 (28.9-35)	0.0015	12.6	A01x8409488	
aboveground biomass-DR	32.2 (31.3-35.3)	2.12	13.6	A01x8682528	
belowground biomass-WW	33.4 (33-36)	0.122	13.8	A01x9090747	
*g*_*s*_ -DR	34.2 (33.4-35.1)	0.022	19.2	A01x9141005	*p* = 0.0269
aboveground biomass-WW	42.8 (40.8-44.5)	3.04	9.3	A01x10838067	
LMA-WW	46.1 (44.6-46.5)	-2.08	18.7	A01x14360906	*p* = 0.039
leaf area-WW	89.7 (86.5-91.3)	1.33	7.4	A01x26133588	
δ13C-DR	89.7 (88.3-91.1)	0.63	23.0	A01x26133588	
*W*_*g*_ -DR	90.5 (86.2-91.3)	2.67	17.1	A01x26378441	
aboveground biomass-WW	91.3 (90.2-92.1)	3.18	10.9	A01x26495518	
*Fv'/Fm'*-WW	91.3 (90.4-92.1)	0.032	24.0	A01x26495518	
height-DR	91.3 (89.6-92.1)	2.56	9.6	A01x26495518	
*A*-WW	91.3 (89.5-92.1)	1.47	11.4	A01x26495518	
*A*-DR	91.3 (89.3-92.1)	1.53	10.6	A01x26495518	
fruit production-WW	91.3 (90.1-92.1)	13.0	39.1	A01x26495518	
*Fv'/Fm'*-DR	91.6 (90.3-91.3)	0.038	20.6	A01x26495518	
fruit production-DR	92.1 (89.7-92.1)	10.2	17.5	A01x26649666	*p* = 0.0464
*g*_*s*_ -WW	92.1 (89.2-92.1)	0.012	10.8	A01x26649666	
δ^13^C -WW	92.1 (86.3-92.1)	0.31	6.4	A01x26649666	
leaf area-DR	92.1 (90.1-92.1)	0.62	5.5	A01x26649666	
Chromosome 2					
*g*_*s*_ -DR	18.6 (14.8-21.2)	-0.014	23.9	A02x1600026	
leaf area-DR	64.9 (49.7-65.3)	0.65	5.9	A02x9399462	
root:shoot ratio-WW	65.3 (52.3-69)	0.0012	8.3	A02x9415100	
*g*_*s*_ -WW	72.7 (71.9-77.3)	0.009	6.0	A02x11479009	
belowground biomass-DR	72.7 (72.3-74.4)	0.048	5.0	A02x11479009	
belowground biomass-WW	76.6 (72.3-78.7)	0.122	12.2	A02x12174045	
aboveground biomass-WW	76.9 (75.6-77.7)	2.30	5	A02x12745444	
leaf area-WW	77.3 (73.1-79)	1.41	7.4	A02x12927010	
Chromosome 3					
belowground biomass-WW	2 (0-8.1)	0.104	8.5	A03x97360	
aboveground biomass-WW	4.5 (2.4-6.2)	2.68	7.0	A03x1233655	*P* = 0.0336
leaf area-WW	4.5 (2.4-8.1)	1.39	7.7	A03x1233655	
days to flowering-WW	9.8 (8.2-14.5)	0.55	8.5	A03x1786910	
height-WW	30.8 (29.9-31.2)	4.75	25.7	A03x5439663	
height-DR	30.8 (29.9-31.2)	2.98	14.8	A03x5439663	
root:shoot ratio-DR	36.7 (32.6-37.9)	-0.0035	9.9	A03x6224923	
days to flowering-WW	67.8 (67-72.8)	0.52	9.2	A03x12997092	
*A*-WW	68.2 (67.9-69.5)	-1.84	16.7	A03x13128260	
*A*-DR	70.5 (66.6-72.7)	-1.29	6.5	A03x13687552	
height-WW	70.5 (68.6-71.1)	-2.44	5.7	A03x13687552	
height-DR	75.2 (74.4-78.1)	-1.94	6.0	A03x14491869	
days to flowering-DR	75.2 (74.4-79.8)	0.57	7.4	A03x14491869	
δ^13^C -WW	76.5 (75.6-77)	-0.63	27	A03x14585658	
δ^13^C -DR	76.5 (75.6-78.1)	-0.81	37.7	A03x14585658	
*Fv'/Fm'*-DR	76.5 (75.6-77.9)	-0.036	22.7	A03x14585658	
*Fv'/Fm'*-WW	76.9 (79.4-81)	-0.039	40.0	A03x14767219	
fruit production-DR	76.9 (75-78)	-25.6	21.5	A03x14767219	
*W*_*g*_-WW	76.9 (75.7-78.1)	-1.07	11.0	A03x14767219	
aboveground biomass-DR	77.7 (75.6-83.9)	-1.94	10.5	A03x14933805	
leaf area-DR	80.2 (74.9-81)	-0.64	4.8	A03x15439617	
belowground biomass-DR	81 (77.7-83.5)	-0.056	8.1	A03x15589868	
leaf area-WW	83.1 (81-86.4)	-1.24	6.6	A03x15983737	
aboveground biomass-WW	86 (83.9-90.1)	-2.48	6.1	A03x16388952	
*Fv'/Fm'*-WW	110.4 (107.4-112.7)	0.022	7.6	A03x21952964	
δ^13^C -DR	115.7 (112.8-116.1)	0.31	4.3	A03x23208268	
belowground biomass-DR	115.7 (113-120.3)	0.046	3.8	A03x23208268	
Chromosome 4					
root:shoot ratio-DR	23.5 (22.3-26.8)	0.0186	4.8	A04x7829955	
δ^13^C -WW	39.2 (36.9-41.3)	-0.27	5.2	A04x12883502	
fruit production-DR	50.8 (49.7-52.8)	18.8	6.9	A04x15025757	
*A*-WW	52.4 (49.6-56)	1.03	5.7	A04x15133897	
Chromosome 5					
*W*_*g*_-WW	19.5 (19.1-28.6)	-1.5	12.1	A05x2841024	
δ^13^C-DR	47.1 (33-47.9)	-0.37	5.8	A05x18948334	
root:shoot ratio-DR	72 (65.6-72.4)	-0.0027	5.3	A05x22970845	
Chromosome 6					
root:shoot ratio-WW	35.2 (30.4-39)	0.0011	7.8	A06x11042216	
height-DR	42.7 (40.7-46.6)	2.10	7.4	A06x16894473	
days to flowering-DR	50.5 (48.1-52.1)	0.48	5.6	A06x19335038	
*g*_*s*_ -WW	53.9 (51.4-57.1)	0.013	12.8	A06x20616311	
δ^13^C -WW	76.2 (72.9-78.7)	-0.27	5.0	A06x22977608	
Chromosome 7					
*g*_*s*_-WW	1.2 (0-3.3)	0.010	7.6	A07x385264	
δ^13^C -DR	16.6 (16-18.2)	-0.52	10.7	A07x9046546	
δ^13^C -WW	16.9 (16.6-19)	-0.51	7.7	A07x9118045	
*g*_*s*_ -DR	20.7 (19.4-23.2)	0.019	15.0	A07x9827612	
days to flowering-DR	28.2 (22.4-29.9)	-0.49	6.3	A07x11888127	
δ^13^C-DR	31.1 (30-32.8)	0.41	6.5	A07x12258663	
*Fv'/Fm'*-WW	31.1 (30.3-33.2)	0.021	6.5	A07x12258663	
*A*-WW	46.5 (43.7-46.9)	0.96	4.8	A07x16000246	
root:shoot ratio-DR	70 (68.8-71.7)	-0.0024	7.7	A07x20416077	
root:shoot ratio-WW	75.8 (75-77.5)	-0.0019	9.7	A07x22133032	
LMA-WW	75.8 (74.6-77.5)	-1.24	9.4	A07x22133032	
Chromosome 8					
LMA-WW	38.6 (35.3-39.8)	-1.17	8.9	A08x16635085	
δ^13^C-WW	52.6 (51.4-54.3)	-0.23	3.8	A08x18475462	
*Fv'/Fm'*-DR	55.5 (54.3-57.1)	-0.017	5.4	A08x18740873	
height-WW	70.6 (63.6-75.5)	-2.13	5.0	A08x19709873	
Chromosome 9					
leaf area-WW	10 (4.6-10.4)	-0.95	3.9	A09x1412906	
days to flowering-WW	27.6 (27.3-30.9)	-0.55	8.8	A09x4597771	
δ^13^C-WW	64.3 (61.4-66.8)	-0.31	6.2	A09x11485309	
*g*_*s*_-WW	66.4 (64.4-68.5)	0.013	11.0	A09x12890329	
*W*_*g*_-WW	67.2 (62.3-69.7)	-0.85	6.5	A09x13903113	
days to flowering-WW	67.6 (65.2-69)	0.49	6.0	A09x14125759	
aboveground biomass-DR	68.9 (68-71.9)	3.15	26.2	A09x14496888	
fruit production-DR	69.3 (68.5-70.3)	31.7	23.0	A09x18372050	*p* = 0.0267
leaf area-DR	69.3 (67.7-70.1)	1.05	12.8	A09x18372050	*p* = 0.0493
aboveground biomass-WW	69.7 (68.6-70.1)	4.08	12.5	A09x18352859	
belowground biomass-WW	70.1 (68.9-72.9)	0.117	10.0	A09x17690478	
leaf area-WW	70.5 (69.1-71.9)	2.24	18.3	A09x17079326	*p* = 0.0091
belowground biomass-DR	71.9 (69.7-74.1)	0.077	11.4	A09x20118570	
fruit production-WW	74.2 (72.6-75.1)	44.4	16.2	A09x22639439	*p* = 0.0377
height-WW	82.8 (80.4-83.2)	2.09	4.9	A09x25757167	
days to flowering-WW	91.4 (90.4-94.1)	0.62	12.5	A09x29142734	
*A*-WW	111.8 (107.7-115.1)	0.87	4.1	A09x32285047	
*g*_*s*_-WW	116.4 (114.3-118)	0.009	5.4	A09x33224026	
leaf area-WW	130.7 (128.9-133.8)	2.08	18.6	A09x34452870	*p* = 0.0075
Chromosome 10					
height-DR	16.9 (12.8-20.3)	1.75	4.7	A10x1631569	
height-WW	17.8 (16.5-20.3)	2.07	4.8	A10x1623273	
*Fv'/Fm'*-WW	18.8 (16.1-20.3)	0.014	4.5	A10x1374109	
*Fv'/Fm'*-DR	28.3 (25.2-36.1)	0.016	4.6	A10x6937636	
fruit production-DR	32.3 (27.3-33.5)	14.6	7.1	A10x7207245	
fruit production-WW	48.3 (46.2-53)	24.8	5.6	A10x11176662	*p* = 0.04
days to flowering-WW	58 (56.3-58.6)	0.54	8.3	A10x12382013	
*g*_*s*_-DR	60 (59.2-61.6)	-0.013	6.2	A10x12574844	
root:shoot ratio-DR	74.1 (72.6-76.1)	0.0024	7.4	A10x13586998	*p* = 0.0091
height-DR	79.9 (76.1-80.7)	-1.82	4.5	A10x14153811	

*Fv'/Fm'* chlorophyll fluorescence in light, *g*_*s*_ stomatal conductance, *W*_*g*_ intrinsic water-use efficiency (*A*/*g*_*s*_), *δ* ^*13*^ *C* carbon isotope composition, *LMA* leaf mass per area

Several traits lacked significant G × E and had strongly positive across-environment correlations (e.g., many gas-exchange traits, Table 3), and correspondingly showed QTL co-localization and similarity of QTL effect across treatments. For example, QTL in WW and DR co-localized for *A* at ~26 cM and ~91 cM on chromosome 1 and ~70 cM on chromosome 3, for *Fv'/Fm'* at ~91 cM on chromosome 1 and ~76 cM on chromosome 3, and for δ^13^C at ~91 cM on chromosome 1, ~76 cM on chromosome 3 and ~17 cM on chromosome 7 (Table 5; Fig. 2); QTL for *g*_*s*_ in WW and DR were found in close cM proximity at the top of chromosomes 1 and 7. Similar to the gas-exchange traits, no G × E was detected for plant height (Table 3), and QTL for plant height co-localized in WW and DR at ~31 cM on chromosome 3 and ~17 cM on chromosome 10. None of the QTL for gas-exchange traits listed above or plant height showed statistically significant environmental interactions (QTL × E, Table 5). Selection acting at QTL that co-localize and have similar magnitude of effect size across treatments would lead to similar phenotypic responses in both well-watered and drought conditions.

Two important yield-related traits (root:shoot ratio, fruit production) had not only significant genotype effects but also significant G × E effects (and hence *r*_*GE*_ < 1), and correspondingly showed some evidence of environment-specific QTL effects. For root:shoot ratio, all nine QTL for this trait were detected in only one environment (that is, all nine had non-overlapping 2-LOD support limits between the DR and WW environments), with one QTL showing a formally significant environmental interaction in ANOVA (at ~74.1 cM on chromosome 10; Table 5; Fig. 2). More generally, other than chromosome 7, each chromosome harbored only one root:shoot QTL, that is, most QTL affecting root:shoot in the two environments were clearly not physically linked and therefore unlikely to be inherited together. For fruit production, we mapped 8 QTL, all of which were either mapped in only one environment or which differed significantly in the magnitude of effect size across environments (QTL × E, Table 5; Fig. 2). However, while the 2-LOD support limits did not overlap, some QTL affecting fruit production in, for instance, DR were in close cM proximity to QTL affecting that trait in WW (e.g., ~69 and 74 cM on chromosome 9 and ~32 and 48 cM on chromosome 10, Table 5), likely leading to common inheritance if multiple causal loci in fact exist. With regard to significant differences in magnitude of effect, a large-effect QTL at ~92 cM on chromosome 1 explained 39 % of the variance for fruit production in WW and 17 % in DR; selection acting at such QTL would result in a similar direction but different magnitude of phenotypic response across different moisture regimes. Other QTL for fruit production are likely to have environment-specific effects, such as that at ~77 cM on chromosome 3, which carries a large effect in DR (22 PVE), has no statistically detectable effect in WW (despite the large effect size in DR and similar *H*^*2*^ of this trait in both environments, Table 1), and is the only fruit production QTL on that chromosome.

QTL for different traits measured within one environment frequently co-localized; at least two or more QTL had overlapping 2-LOD support intervals at 18 different chromosomal locations. In particular, large blocks of QTL co-localized at four specific regions: a QTL affecting 6 traits was mapped between 27-34 cM chromosome 1, a QTL affecting 9 traits mapped between 90-92 cM on chromosome 1, a QTL affecting 10 traits mapped between 75-86 cM on chromosome 3, and a QTL affecting 8 traits mapped between 64-74 cM chromosome 9 (Table 5; Fig. 2). In these locations, the direction of additive effects was consistent with patterns of genetic correlations among traits, suggesting pleiotropy or close linkage of causal genetic loci for multiple traits (Table 5; Fig. 2). With regard to functional relationships and specific traits, QTL often affected more than one aspect of gas-exchange (most notably at the bottom of chromosome 1 and 75-86 cM on chromosome 3; Table 5; Fig. 2). Many aspects of gas-exchange were also negatively genotypically correlated with flowering time (Table 4), and correspondingly QTL with joint effects on phenology and physiology were identified in several regions, including ~67-76 cM on chromosome 3 (for days to flowering, *A*, *Fv'/*Fm', and δ^13^C under both DR and WW), ~28-31 cM on chromosome 7 (for days to flowering and δ^13^C under DR), ~64-67 cM on chromosome 9 (for days to flowering, *g*_*s*_, *W*_*g*_, and δ^13^C under WW). In each of these cases (among gas-exchange traits or between gas-exchange traits and phenology), selection on one trait could lead to a response in a second trait due to QTL correlations.

## Discussion

Water availability is increasingly unpredictable, and expected to decline in many areas globally [62], which has implications for both the stability of crop yield and the evolution of plants in natural populations. In this study, our goal was to characterize the genetic architecture of multiple drought-response strategies including: 1) which traits are responsive to and maximize yield under season-long field drought in *B. rapa*, 2) if different or similar genotypes perform best in drought and well-watered conditions, 3) whether moisture status in the field affects the magnitude and direction of genetic correlations between drought-response traits, and 4) patterns of QTL effects across water regimes, including allelic contributions from divergent parental genotypes. We exposed genotypes of *B. rapa* to well-watered and drought conditions in a field setting, with the drought treatment being of sufficient strength to reduce below-ground biomass by 31 % and above-ground biomass and fruit production both by 48 %. Drought also resulted in increased relative root:shoot ratios and intrinsic water-use efficiency. Phenology and gas-exchange traits exhibited little genotypic variation in drought response, while root:shoot allocation and yield showed both G × E and QTL × E interactions. Further, most bivariate genotypic and QTL correlations involving phenology and gas-exchange were similar across environments, whereas those involving longer-term allocation responses were more variable across environments. The results suggest a stronger effect of altered allocation on performance under drought than of either phenological or gas-exchange responses. Below we discuss the results in light of adaptive drought-response strategies and the implications for crop improvement.

### Mechanisms of drought response

Plant growth theory predicts that when plants are stressed by a lack of resources, relative allocation belowground increases while total biomass production declines [63]. The decline in production occurs through all or some combination of declines in leaf-level photosynthesis and in leaf area and greater relative allocation to roots even if absolute root growth is less [64, 65]. These shifts in gas-exchange and biomass allocation are often associated with improved survival in drought [12, 13, 66]. At the genotypic level, a prior greenhouse study [24] that used a subset of the same genetic lines of *B. rapa* observed that genotypic water use efficiency (*W*_*g*_) varied dramatically across drought and well-watered conditions, and the ability of genotypes to optimize *W*_*g*_ to their environment was very important for overall performance (i.e., greater *W*_*g*_ was associated with greater biomass in drought, whereas lower *W*_*g*_ was associated with better performance in well-watered conditions). This previous study did not measure fruit production, the primary yield component of oilseed crops. In another recent study in *B. rapa* using different parental genotypes, El-Soda et al. [67] similarly observed that reduced stomatal conductance was advantageous under drought conditions.

Gas-exchange and resource partitioning responses to drought in the current study differed from past studies in this species, and suggest a more important role for biomass partitioning than for either gas-exchange or phenology as a mechanism of drought response. Although all components of yield for this species (i.e., above-ground and below-grown biomass and fruit production) were reduced by drought, δ^13^C and other gas-exchange traits were not strongly affected by water regime or by the interaction of genotype × water regime. Consistent with the lack of G × E interactions, correlations involving gas-exchange traits were similar across the two water regimes, including for instance that greater values of *A*, *Fv'/Fm'*, *g*_*s*_ and δ^13^C were always associated with improved yield (fruit production) (Table 4). Flowering time, which may accelerate in response to drought and lead to drought avoidance in some species under season-long drought conditions similar to those here [14], was likewise unresponsive to water regime in the current study (Table 3), and was unrelated to fruit production under either well-watered or drought conditions (Table 4).

We observed that root:shoot ratio was affected by water regime and by the interaction of genotype × water regime, and most bivariate correlations involving root:shoot ratio differed significantly across treatments. In well-watered conditions, root:shoot ratio was uncorrelated with traits related to photosynthesis (*A, Fv'/Fm'*, δ^13^C, and LMA) and significantly positively correlated with overall size (above- and below-ground biomass), while in drought, genotypes that had the greatest allocation to roots had lower values for all of these traits as well as lower fruit production. With regard to yield in drought, greater values of root:shoot are unlikely to reflect poor overall genotypic vigor, because greater values of this trait were observed in larger than average genotypes in well-watered conditions. Instead, under prolonged, season-long drought, moderate increases in root:shoot ratios above those observed under well-watered conditions (Fig. 1) may increase survival (and almost all plants in the drought treatment in fact survived to reproduce) and ensure sufficient water supply for photosynthesis, whereas increasing root:shoot beyond a level required to ensure survival and favorable water relations may result in yield tradeoffs. This explanation is supported by the fact that after statistically factoring out stomatal conductance (*g*_*s*_), photosynthesis (*A*) remained negatively related to root:shoot (Fig. 1b, inset); low-to-moderate increases in root:shoot observed under drought therefore provided water supply sufficient to sustain maximal photosynthesis levels similar to those observed under well-watered conditions (Fig. 1b), while the greatest investment in roots may have involved both a negative feedback on photosynthesis [68] and a cost in terms of fruit production. In sum, these results suggest an optimum value exists for root:shoot under drought, reflecting the balance between increasing allocation to improve survival and limiting allocation to avoid yield tradeoffs (see below for relevance to crop improvement).

With regard to generating a synthetic understanding of drought adaptation, it is worth considering both the observed similarities and the possible source of differences between the current and past studies. Both the current study and earlier ones identified genotypes that had high biomass accumulation across all moisture treatments, indicating that some high-fitness or high-yield genotypes exist that perform well regardless of the severity of drought or exact experimental (greenhouse *vs*. field) setting. With regard to divergence among studies in the traits associated with performance, differences in the water-holding capacity of the soil used across experiments likely affected the severity of drought experienced by plants and reduced G × E interactions for gas exchange in the current study. That said, because field soil was utilized in the current experiment, the treatment is likely reflective of the effective drought and potential soil heterogeneity that crop plants experience in the field. Large genotypic differences in average water use segregating in the complete population may have also outweighed genotypic variation in drought responsiveness, which has been observed between species [69]. Finally, plants in the current study were germinated in a greenhouse and then transplanted to the field, and this transplanting step may have an unquantified effect on results. Regardless of proximate experimental explanations for the differences in the relative importance of gas-exchange *vs*. phenology *vs*. biomass partitioning traits, the level of drought imposed in the current study can be considered agronomically relevant because the drought reduced yield as estimated from all the three targets of harvest for this crop species (i.e., above-ground biomass of relevance to leaf crops, below-ground biomass relevant to root crops, and fruit production the target in oilseed crops). Under these conditions, shifts in root allocation in response to drought seemingly enable favorable water relations and have pronounced effects on yield. Future studies could profitably test how the duration and/or severity of drought affect the adaptive value of different drought-responsive traits.

### Implications for crop improvement

Because the cultivation conditions in the current study do not fully reflect standard agricultural practices (i.e., seedlings were transplanted in this study instead of being field sown and were spaced farther apart than in agricultural settings), some results must be interpreted with caution; nevertheless, several important relationships among traits may be relevant for future crop improvement. Both gas-exchange traits and root:shoot ratio were associated with yield. The magnitude of the genotype and QTL effects as well as across-environment associations for gas-exchange, root:shoot, and yield is relevant to crop improvement efforts for drought resistance within this species. First, considering traits individually, selection for greater gas-exchange among genotypes similar to those of the current experimental population (or select alleles from the parental genotypes) will result in greater values of gas-exchange in well-watered and drought conditions in the field, as evidenced by the large magnitude of the genotype effects and of the across-environment correlations (Table 3, *r*_*GE*_ for *A*, *Fv'/Fm'*, *g*_*s*_, δ^13^C). Consistent with genotypic patterns, QTL typically affected gas-exchange traits in both environments. The strong genotypic and QTL correlations across the moisture treatments applied here suggest limited opportunity for environment-specific evolution and targeted optimization of gas-exchange under moderate season-long drought. While preserving greater *A* across environments is desirable, greater values of *g*_*s*_ across moisture environments may be disadvantageous if increased values of this trait reduce survival under drought (although in the current study little mortality was observed and modulation of root:shoot seemingly maintained favorable water relations for photosynthesis under drought). Allocation shows greater opportunity for independent evolution across moisture settings and targeted improvement under drought, as *r*_*GE*_ was lower (Table 3) and few QTL were identified with clearly common effects on root:shoot across environments.

The behaviors of gas-exchange and allocation (and other unmeasured phenotypes) are ultimately determinants of whole-plant traits such as fruit production, which are of agronomic value or important to fitness of wild genotypes. Fruit production showed strong genotypic (Table 3) and QTL (Fig. 2) associations across environments. Although several fruit production QTL had significant environmental interactions, these interactions arose from differences in magnitude of effect (often shifting between moderate to large percent variance explained) rather than differences in sign or in presence *vs*. absence of effect across environments. Selection acting at such QTL would have parallel effects on yield albeit of somewhat different magnitude across environments. Identifying genotypes and QTL alleles that confer comparatively greater yield under well-watered conditions and preserve yield under drought has utility in stabilizing crop yield.

Aside from across-environment relationships for individual traits, observed genotypic and QTL correlations among diverse traits within environments affect the opportunity for crop improvement. Not surprisingly, many gas-exchange traits were strongly correlated within the well-watered and within the drought treatment and will likely exhibit correlated selection responses during crop improvement as a consequence of mechanistic connections between, for instance, CO_2_ supply (estimated as *g*_*s*_) and *A* as well as *Fv'/Fm'* and *A*. Also relevant for crop improvement is the fact that root:shoot was negatively associated with yield, but only in drought (as described above). With regard to agronomically desirable allocation patterns under drought, an intermediate optimal value may exist for root:shoot that reflects a balance between the positive effect that increased root:shoot has on survival under drought *vs*. the negative impact of increased root allocation on fruit production. Our results in fact suggest it may be possible to select genotypes with moderate root:shoot that will survive and maintain greater levels of *A* under drought with minimal negative impact on yield (circled genotypes in Fig. 1b inset).

As in *Arabidopsis* [70] and an earlier study in *B. rapa* [24, 41], genotypic correlations were observed between gas exchange (*A* and *Fv'/Fm'*) and flowering time, such that delayed flowering was associated with decreased photosynthesis under both water regimes used in the current study. Multiple QTL contributed to these genotypic correlations within both environments. This association may pose an important constraint on crop improvement, if selection for an optimal (delayed) flowering time within a given region of cultivation leads to reductions in photosynthesis. More specifically under drought, early flowering was associated with increased stomatal conductance, *g*_*s*_ (Table 4). This association counters a coordinated and adaptive drought-response syndrome, because while early flowering may enable “drought escape”, increased stomatal conductance counters a “dehydration avoidance” strategy [9–11]. However, one caveat to these results is that because gas-exchange measurements were taken at flowering and occurred over a period of several weeks, it is possible that we observed this correlation because of progressive changes in photosynthesis occurring as plants aged or in response to changes in environmental conditions that occurred as the growing season progressed. Also, because of the time required to take the gas-exchange measurements in this study, some measurements were taken several days past flowering which may have affected this correlation. That being said, variation in measurement dates relative to flowering within genotypes would likely lead to higher within-genotype variance and reduced accuracy in estimation of the genotypic values, which would likely reduce the strength of trait associations, rather than leading to false positive associations. In sum, it is possible that the measurements of these traits may involve some measurement error. Further experiments are necessary to strengthen our understanding of the relationship between flowering time and photosynthesis in *B. rapa* in field conditions.

Also relevant for crop improvement is the identification of QTL that are consistently expressed across experiments and environments. We compared the position and additive effects of QTL identified in the present study to those identified in previous studies using the same RILs, but from different environments (i.e., [37, 38, 40, 41, 71]). Overall, only a small proportion of the QTL found in this study were identified in previous studies, indicating that the environment in which the plant is grown can strongly affect the loci underlying phenotypic trait variation. In total, we identified 10 QTL for a range of traits that were previously identified in past growth chamber experiments. Several QTL for ecophysiological were previously identified in [41], namely QTL for *g*_*s*_ on the top of chromosome 1 in both treatments; a QTL at the bottom of chromosome 3 for *Fv'/Fm'* in WW, and a QTL at 53 cM on chromosome 8 for *δ*^*13*^*C* in WW. Several QTL were previously identified for vegetative and allocation traits in [40], namely QTL in the center of on chromosome 3 for biomass traits in both treatments, and a QTL at 70 cM on chromosome 7 for root:shoot in both treatments. Finally, a QTL in the center of chromosome 3 for plant height in both treatments was previously identified in [37]. For all of these QTL, the r500 allele had a more favorable effect on the trait, suggesting that selection for r500 alleles at these QTL would likely have a positive effect on trait values regardless of the environment in which plants are grown. Although we also compared the positions of QTL identified in the present study to those identified in the field using different lines of the same species [67], no common QTL were identified, likely because of different causative alleles segregating in the two mapping populations.

With further regard to opportunities for crop improvement and the parental genotypes used here, varieties domesticated for oil production often express greater values of gas-exchange, and specifically greater values of *A*. Cultivars of short-season soybean (*Glycine max*) show improvement of oil yield in combination with increases in photosynthetic rates [72, 73], and oil production in *Helianthus annuus* is also positively correlated with a proxy for photosynthetic rates [74]. More closely related to the current study species, photosynthesis is known to affect oil production in modern rapeseed oil, *Brassica napus* [75–77]. In the current study, we found that the oilseed parent, R500, contributed most but not all of the positive-effect alleles at QTL for all gas-exchange traits; the contribution of some positive-effect alleles for these traits from the “weedy” parent (imb211) is consistent with potential crop improvement by introduction from wild/landrace lines.

## Conclusions

In contrast to the results of previous studies, the results of the current study suggest a stronger effect of altered biomass allocation on performance under drought conditions in the field than either phenological or gas-exchange responses. Here, we found that root:shoot ratio had an environment-specific relationship with photosynthesis, biomass and yield; root:shoot ratio was uncorrelated with traits related to photosynthesis and significantly positively correlated with overall size in well-watered conditions, whereas in drought, genotypes that had the greatest allocation to roots had lower photosynthesis, biomass, and fruit production. Under prolonged, season-long drought, plants that demonstrate moderate increases in root:shoot ratios above those observed under well-watered conditions may have increased chances for survival and a sufficient water supply for photosynthesis, whereas increasing root:shoot beyond a level required to ensure survival and favorable water relations may result in yield/fitness tradeoffs. Crop improvement efforts may focus on selecting genotypes with an intermediate optimal value for root:shoot in drought that reflects a balance between the positive effect that increased root:shoot has on survival while avoiding the negative impact of increased root allocation on fruit production. Our results in fact suggest it may be possible to select genotypes with moderate root:shoot that will survive and maintain greater levels of photosynthesis under drought with minimal negative impact on yield. Overall, these results indicate that biomass partitioning may have a particularly important role in drought response in the field; future crop improvement efforts in a diverse range of species that aim to increase yield across a range water regimes may be well served to investigate the environmental dependency of the effects of biomass partitioning on yield.

## Additional file

Additional file 1: Phenotypic dataset for each individual in both drought and well-watered conditions. (XLS 1770 kb)

## Abbreviations

*A*: Photosynthetic rate
ANOVA: Analysis of variance
BLUP: Best linear unbiased predictor
DR: Drought
cM: Centimorgans
G × E: Genotype by environment interaction
*Fv'/Fm'*: Chlorophyll fluorescence in light
FDR: False discovery rate
*g*_*s*_: Stomatal conductance
GLM: Generalized linear model
*H*^*2*^: Broad-sense heritability
IRGA: Infrared gas analyzer
LMA: Leaf mass per area
*r*_*GE*_: Cross-environment genetic correlation
lod: Logarithm of odds
PVE: Percent variance explained
QTL: Quantitative trait locus
QTL × E: Quantitative trait locus by environment interactions
RILs: Recombinant inbred lines
SNP: Single nucleotide polymorphism
VWC: Volumetric water content
V_G_: Among-genotypic variance
V_G_/V_P_: Among-genotypic variance divided by total phenotypic variance
*W*_*g*_: Intrinsic water use efficiency (*A*/*g*_*s*_)
WW: Well watered
δ^13^C: Carbon isotope composition
